# Protective efficacy of influenza group 2 hemagglutinin stem-fragment immunogen vaccines

**DOI:** 10.1038/s41541-017-0036-2

**Published:** 2017-12-15

**Authors:** Troy C. Sutton, Saborni Chakraborty, Vamsee V. A. Mallajosyula, Elaine W. Lamirande, Ketaki Ganti, Kevin W. Bock, Ian N. Moore, Raghavan Varadarajan, Kanta Subbarao

**Affiliations:** 1 Laboratory of Infectious Diseases, NIAID, NIH, Bethesda, MD USA; 2 Molecular Biophysics Unit, Indian Institute of Science, Bangalore, Karnataka India; 3 Comparative Medicine Branch, Infectious Disease Pathogenesis Section, NIAID, NIH, Bethesda, MD USA; 4 Present Address: WHO Collaborating Centre for Reference and Research on Influenza at the Peter Doherty Institute, 792 Elizabeth Street, Melbourne, VIC Australia

## Abstract

The stem of the influenza A virus hemagglutinin (HA) is highly conserved and represents an attractive target for a universal influenza vaccine. The 18 HA subtypes of influenza A are phylogenetically divided into two groups, and while protection with group 1 HA stem vaccines has been demonstrated in animal models, studies on group 2 stem vaccines are limited. Thus, we engineered group 2 HA stem-immunogen (SI) vaccines targeting the epitope for the broadly neutralizing monoclonal antibody CR9114 and evaluated vaccine efficacy in mice and ferrets. Immunization induced antibodies that bound to recombinant HA protein and viral particles, and competed with CR9114 for binding to the HA stem. Mice vaccinated with H3 and H7-SI were protected from lethal homologous challenge with X-79 (H3N2) or A/Anhui/1/2013 (H7N9), and displayed moderate heterologous protection. In ferrets, H7-SI vaccination did not significantly reduce weight loss or nasal wash titers after robust 10^7^ TCID_50_ H7N9 virus challenge. Epitope mapping revealed ferrets developed lower titers of antibodies that bound a narrow range of HA stem epitopes compared to mice, and this likely explains the lower efficacy in ferrets. Collectively, these findings indicate that while group 2 SI vaccines show promise, their immunogenicity and efficacy are reduced in larger outbred species, and will have to be enhanced for successful translation to a universal vaccine.

## Influenza: Developing a universal vaccine for influenza A group 2

Progress has been made towards a universal vaccine targeting the ‘group 2’ subtype of influenza A virus. Today’s flu vaccines target the ‘head’ section of the virus’ hemagglutinin protein; however, this section acquires mutations which require the reformulation of vacccines. In this paper, a vaccine candidate designed to focus an immune response against the more stable protein ‘stem’ is described by a team of scientists led by Kanta Subbarao of the United States’ National Institutes of Health and Raghavan Varadarajan of the Indian Institute of Science. The candidate vaccine offered moderate protection to mice but did not provide significant antiviral effects when tested in ferrets. The authors suggest that, while their approach shows promise, improvement is needed before it could be translated into vaccines against human influenza infection.

## Introduction

Each year seasonal influenza epidemics cause 3–5 million cases of severe illness and more than 250,000 deaths worldwide.^1^ In the event an antigenically novel virus emerges and initiates a pandemic, the disease burden can be much greater.^2^ Vaccination is the most effective control strategy for influenza. Due to antigenic drift and strain mismatch, the efficacy of seasonal influenza vaccines varies from 23–85%,^1,3^ and these vaccines confer little or no protection against pandemic influenza viruses.

The major target of licensed influenza vaccines is the viral hemagglutinin (HA) protein. The influenza A HAs are phylogenetically divided into group 1 encompassing H1, H2, H5, H6, H8, H9, H11, H12, H13, H16, H17, and H18, and group 2 including H3, H4, H7, H10, H14, and H15 subtypes.^4^ The HA is expressed on the virion as a trimer in the pre-fusion state (HA0), and is cleaved by cellular proteases to HA1 and HA2.^5^ Most of the HA1 chain forms the globular head of the HA and this is the immunodominant portion of the HA. Licensed vaccines induce neutralizing antibodies against the HA head; however, mutations in the globular head lead to immune escape and reduced vaccine efficacy. In contrast, the HA2 region is highly conserved and forms most of the stem (or stalk) of the HA. Importantly, recent efforts to define the influenza-specific antibody repertoire have identified low levels of broadly neutralizing antibodies (bnAbs) that bind the HA stem in previously infected and vaccinated individuals.^6–11^ As a result, the HA stem region has been identified as an ideal target for the development of broadly protective or “universal” influenza vaccines.

To target the HA stem, several strategies including vaccination with chimeric HA, headless HA proteins,^12–15^ and polypeptides mimicking the HA stem^16–21^ have been evaluated in animal models. These approaches have been developed against group 1 viruses and have shown protection in mice, ferrets, and non-human primates;^12,13,16–22^ however, there is limited cross-group (i.e. group 2) protection. This is most likely due to a restricted footprint of conserved residues,^8,23^ and structural or biochemical differences between group 1 and group 2 HAs.^24^ We previously designed group 1 stem immunogens (SI) that mimic the native, pre-fusion conformation of the HA stem and focus the antibody response to this region.^13,16–19,21,25–27^ Herein, we applied this approach to develop SI that encompass the epitope targeted by bnAbs that bind group 2 HA and developed SIs derived from the H3 and H7 HA stem. Biophysical characterization indicated the group 2 SI form folded trimeric proteins, and SI vaccination induced stem-directed antibodies that protected from lethal homologous and intrasubtypic challenge, and resulted in moderate protection against lethal heterologous challenge in mice. In ferrets, group 2 SI vaccination induced relatively low levels of HA stem-directed antibodies that did not reduce clinical disease but were associated with a non-significant reduction in viral shedding by ~30-fold on day 5, following robust H7N9 virus challenge. These findings indicate that while vaccination with group 2 SI induces robust protective immunity in mice, further optimization is needed to enhance immunogenicity and protection in ferrets.

## Results

### Design of vaccine immunogens targeting the glycan-masked, pan-influenza neutralizing epitope of the group 2 HA stem

**Fig. 1:**
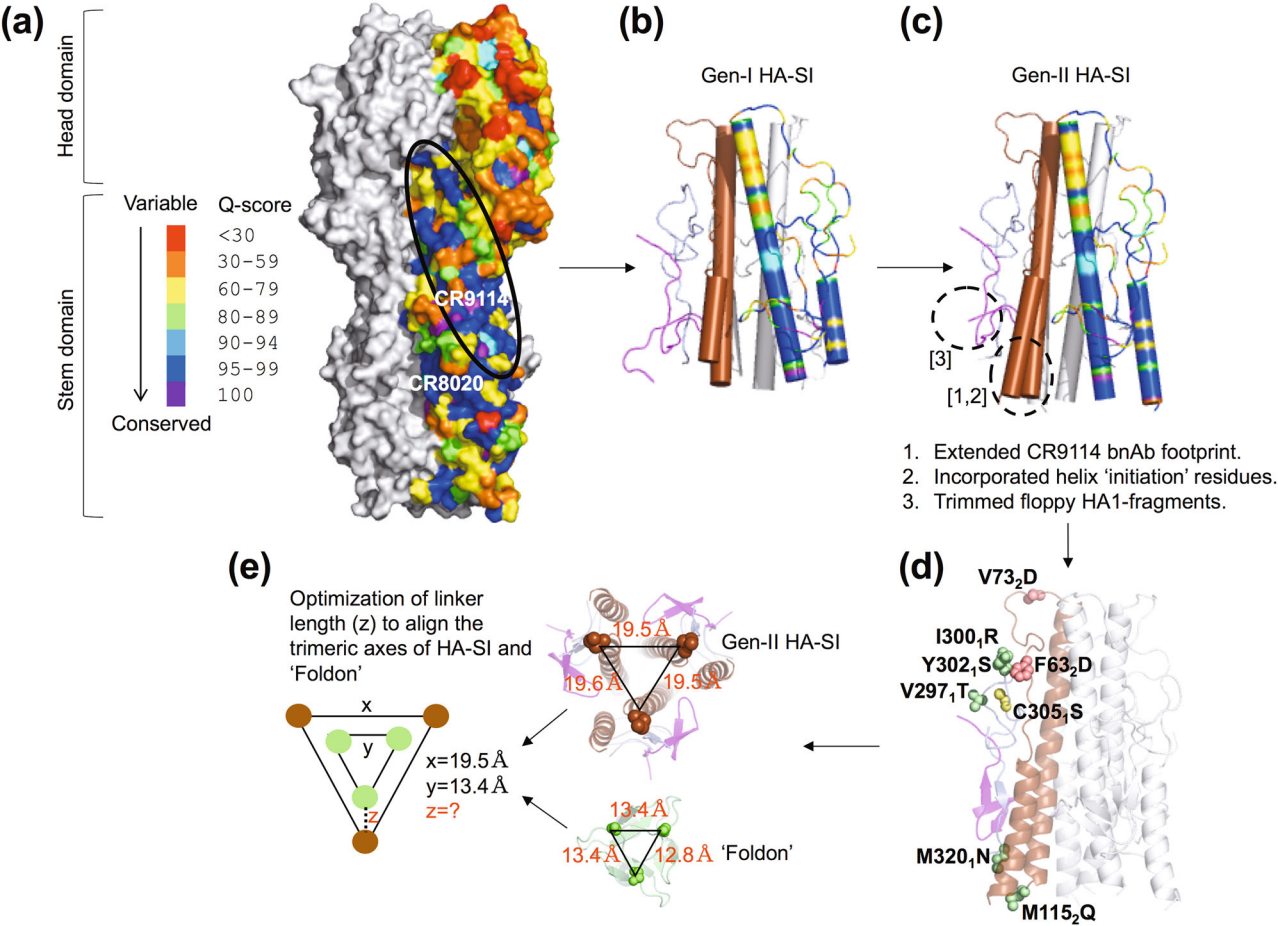
Design of “headless” HA-SI **a** The conserved, non-overlapping epitopes targeted by HA stem-directed bnAbs (CR9114, CR8020) are shown. The sequence conservation across all full-length influenza A Group 2 HA sequences (*n* = 5462 for H3N2, and *n* = 55 for H7N9) determined for viruses isolated from human hosts was mapped onto a monomer of the surface representation of H3 HA A/Hong Kong/1/68 (PDB ID: 1HGD). Rest of HA (light gray). **b** Our first generation of Group 2 HA-SI targeting the α-helix contained only ~50% of the pan-influenza bnAbs epitopes. A monomer is colored according to the indicated conservation score. The individual HA stem fragments are colored distinctly in another monomer (19_1_–46_1_ (magenta), 290_1_–321_1_ (light blue), 44_2_–113_2_ (brown)). Rest of HA-SI (light gray). **c** The interaction network within the HA stem was re-evaluated to extend the pan-influenza bnAb epitope footprint (~90%) in the second generation of Group 2 HA-SI (cartoon). A monomer is colored according to the conservation score. The individual HA fragments constituting the HA-SI are colored distinctly in another monomer (24_1_–46_1_ (magenta), 290_1_–322_1_ (light blue), 37_2_–115_2_ (brown)). Rest of HA-SI (light gray). **d** Mutations were rationally engineered in HA-SI (cartoon) to mask exposed hydrophobic patches (space-fill, light green). Aspartate mutations (F63_2_D and V73_2_D) were incorporated in the β-loop (HA2-subunit) to destabilize the extended, coiled-coil conformation of HA (space-fill, salmon). C305_1_ was mutated to Ser to prevent the formation of protein aggregates with incorrect, intermolecular disulfide bonds (space-fill, yellow). **e** The distance between the C-terminal residue (M115_2_Q) of the HA stem fragment (37_2_–115_2_) (PDB ID: 1HGD) and the N-terminal residue (*G*_1_) of foldon (PDB ID: 1RFO) was calculated to determine the optimal linker length

To engineer immunogens targeting the group 2 HA stem, sequence conservation analysis of full-length group 2 HAs delineated conserved, non-overlapping epitopes that are targeted by distinct bnAbs (Fig. 1a). The α-helix (HA2-subunit) is targeted by the pan-influenza bnAbs CR9114 and FI6;^8,23^ while the viral membrane proximal β-sheet constitutes the epitope for heterosubtypic group restricted bnAbs CR8020 and CR8043.^28,29^ In previous studies, we developed a first generation group 2 HA SI that conferred partial protection (40–50%) against homologous virus challenge in mice.^19^ As this SI contained only ~50% of the epitope of the pan-influenza bnAbs FI6 and CR9114 (Fig. 1b), to improve protection we expanded the footprint to incorporate ~90% of the epitope (Fig. 1c) and introduced mutations to mask hydrophobic patches of the HA stem (Fig. 1d). With this approach we generated a second-generation SI from the HA of A/Hong Kong/1/1968 (H3N2) termed HkH3HA10v2F,^18,19^ however, for brevity the SI is now designated Hk68-H3-SI.

Although heterologous full-length HAs share an overall coarse-grained structural similarity, specific differences at the atomic level make translation of the design framework to non-identical influenza virus strains challenging. Therefore, we evaluated the generality of our design protocol by generating SI from the highly divergent, heterologous A/Philippines/2/1982 (H3N2) (Supplementary Table S1), and the heterosubtypic A/Shanghai/1/2013 (H7N9) viruses (Supplementary Table S1). These SI were designated Ph82-H3-SI and Sh13-H7-SI, respectively; the SI sequences are listed in Supplementary Fig. S1. Importantly, the development of an array of SIs enabled evaluation of the landscape of protective immunity conferred by these immunogens.

### Characterization of soluble recombinant SIs

SIs were engineered to be expressed in *Escherichia coli* ^30^ and were purified to homogeneity from the soluble fraction of the culture lysate via single-step affinity chromatography. The HA stem is characterized by the presence of extensive α-helical stretches. Consistent with this, the averaged circular dichroism (CD) spectra of SIs were indicative of a well-folded protein rich in α-helices (Supplementary Fig. S2a). The conformation of SIs was also probed using intrinsic tryptophan fluorescence measurements. A red-shift in the fluorescence emission maxima of SIs upon denaturation with guanidine hydrochloride indicated burial of hydrophobic residues under native conditions, suggesting a well-packed, folded conformation (Supplementary Fig. S2b). The thermal stability of the SIs was also estimated using differential scanning fluorimetry, wherein the intrinsic protein fluorescence was monitored in parallel at 330 nm and 350 nm as a function of temperature. The high transition-midpoints (*T*_m_) of thermal unfolding for SIs suggest that the designed proteins are resistant to thermal stress (Supplementary Fig. S2c). We next determined the oligomeric state of SIs by analytical gel-filtration chromatography under native conditions. Encouragingly, the SIs eluted exclusively as trimers in solution (Supplementary Fig. S2d). The lack of higher-order aggregates in solution suggests resurfacing of exposed hydrophobic patches mitigates aggregation.

### Rational protein engineering leads to minimal perturbation of antigenic sites

Binding assays using conformation dependent HA-stalk antibodies are a sensitive probe of HA conformation. Therefore, we evaluated binding of CR9114 and FI6 IgG to the SI. By direct enzyme-linked immunosorbent assay (ELISA), both antibodies exhibited dose-dependent binding to all three SIs **(**Fig S2 i–j **)**. Furthermore, when biolayer interferometry (BLI) was used to measure binding of the SI to CR9114, both the Hk68-H3-SI and Ph82-H3-SI exhibited high-affinity binding (Supplementary Table S2, Supplementary Fig. S2 e–h), with equilibrium dissociation constants (*K*_D_) of 1.5 ± 1.43 nM and 3.30 ± 1.27 nM, respectively. Surprisingly, the Sh13-H7-SI bound CR9114 with significantly lower affinity (*K*_D_: 127 ± 1.4 nM), owing to a higher off-rate ((*k*_off_ (1/s): 1.31 ± 0.06 × 10^−3^) (Supplementary Fig. S2h). Further investigation is necessary to discern the basis of the unusually high off-rate observed for Sh13-H7-SI, although the high-affinity binding to HA stem-directed bnAbs further confirms the structural integrity of the SIs.

### Group 2 SI vaccination of mice induces high levels of stem-directed antibodies that compete for binding with CR9114 to the HA stem

**Fig. 2:**
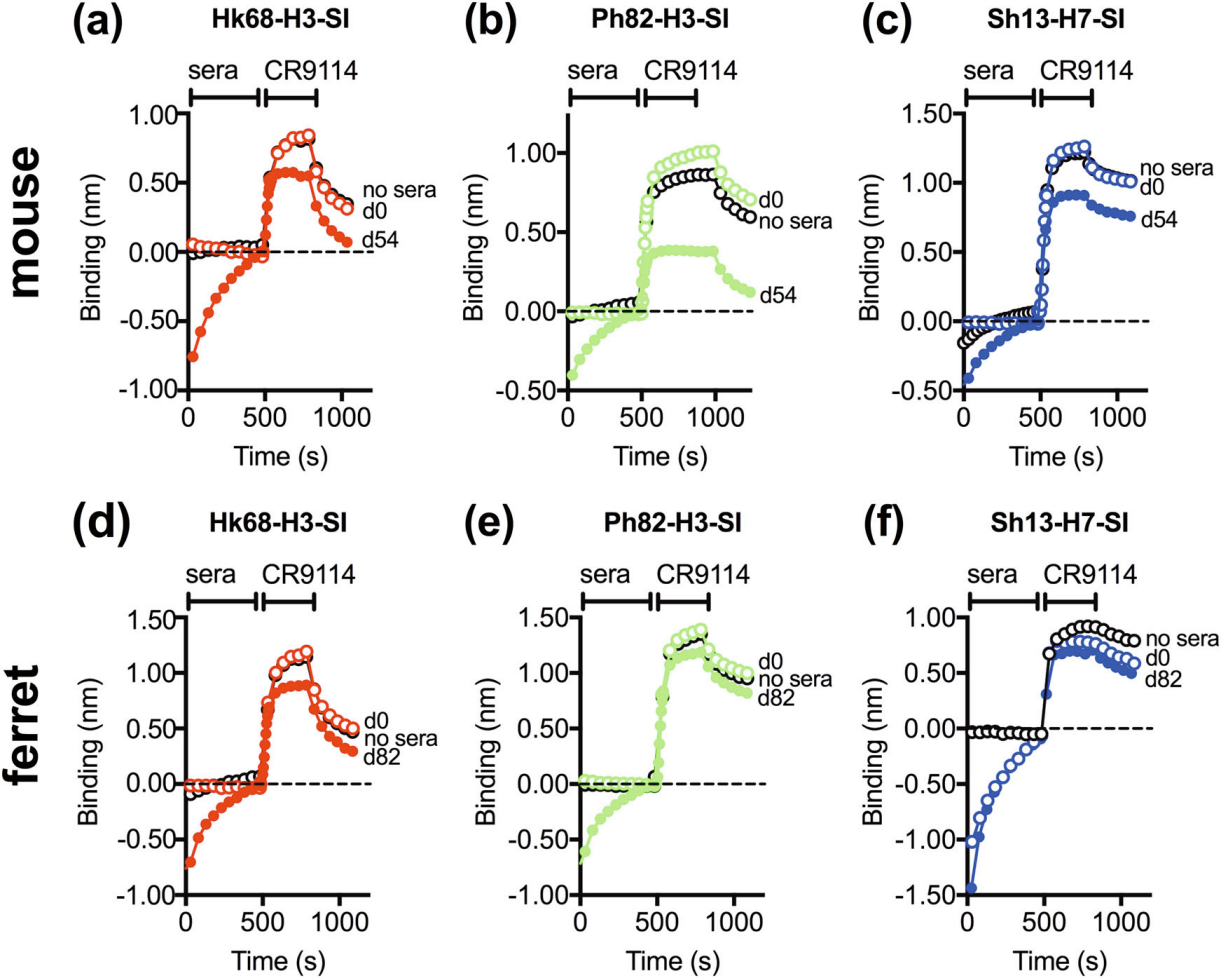
SI vaccination of mice and ferrets induces antibodies that competitively inhibit the binding of CR9114 to full-length recombinant HA. Competitive binding to full-length recombinant HA of post-vaccination mouse and ferret sera with the broadly neutralizing monoclonal antibody CR9114. Panels **a** and **b** display competitive binding of Hk68-H3-SI and Ph82-H3-SI mouse anti-sera to recombinant H3 HA A/Hong Kong/1/1968, and **c** displays competition binding of CR9114 and Sh13-H7-SI mouse anti-sera against recombinant H7 HA A/Anhui/1/2013 (H7N9). Panels **d** and **e** display competitive binding of ferret Hk68-H3-SI and Ph82-H3-SI anti-sera to recombinant H3 HA A/Hong Kong/1/1968, respectively, and **f** displays competition binding of CR9114 and Sh13-H7-SI ferret anti-sera against recombinant H7 HA A/Anhui/1/2013 (H7N9). Within group sera for each species were pooled (*n* = 13/group for mice, *n* = 4/group for ferrets) and three independent competition assays were performed. Shown are representative traces from one assay. Day 0 sera is naive pre-vaccination sera, and d54 and d82 represents post-vaccination sera in mice and ferrets, respectively

**Fig. 3:**
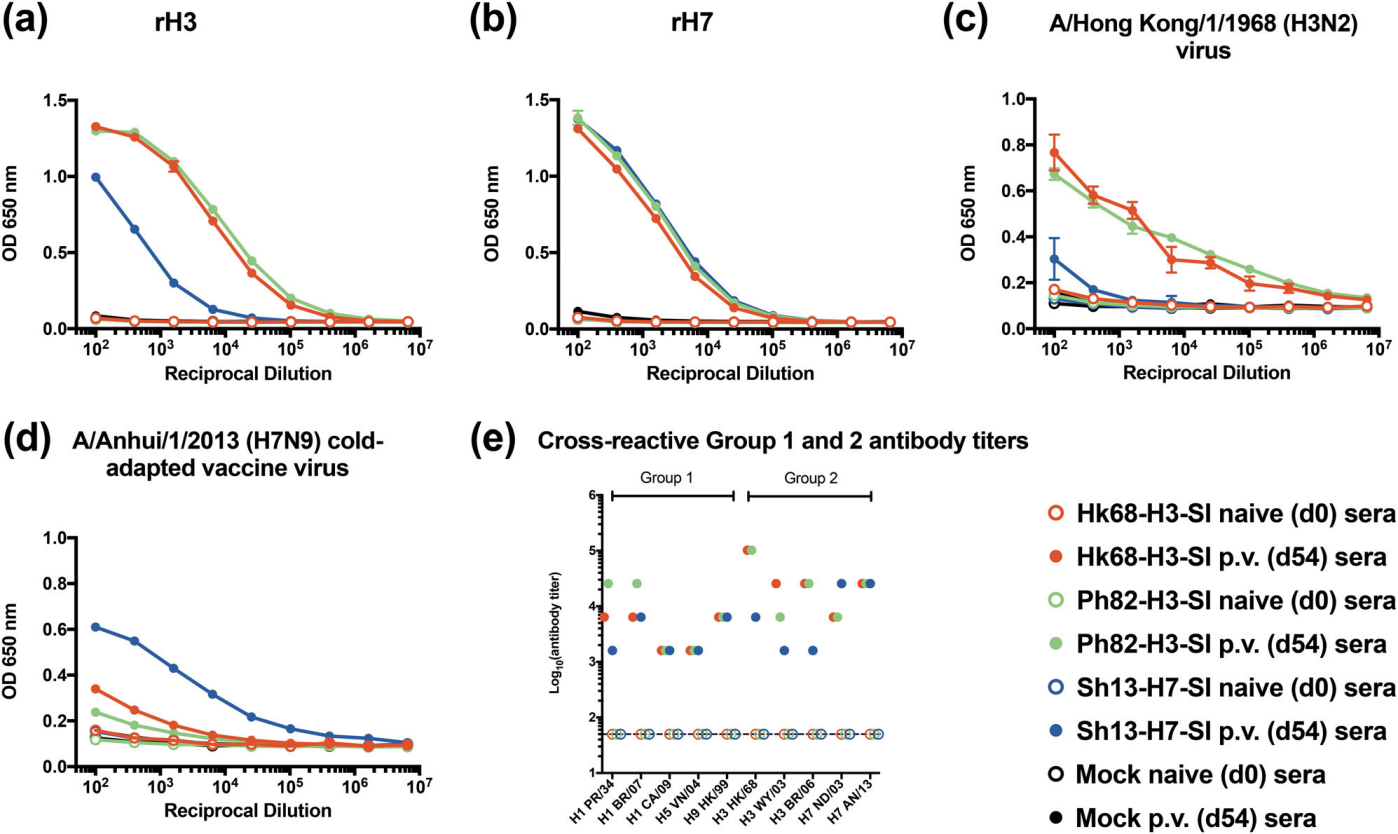
SI vaccination of mice induces high titers of stem antibodies that bind a breadth of group 1 and 2 recombinant HA proteins, and that bind HA on viral particles. Binding antibody titers determined by ELISA in mouse sera using recombinant HA and whole virus as coating antigens. Antibody titers against recombinant **a** H3 HA from A/Hong Kong/1/1968 (H3N2) and **b** H7 HA from A/Anhui/1/2013 (H7N9). Panels **c** and **d** displaying binding antibody titers against purified wild-type A/Hong Kong/1/1968 (H3N2) influenza virus, and A/Anhui/1/2013 (H7N9) cold-adapted vaccine virus, respectively. ELISA assays were optimized with pooled mouse sera (due to limited volume of sera) (*n* = 13/group) and subsequently performed in triplicate. Results are expressed as mean ± s.e.m. **e** Antibody binding titers were determined in mouse sera against a range of different full-length recombinant HA proteins. Assays were performed in duplicate in two independent experiments and mean values from all experiments are plotted. Shown are the end-point titers for pre and post-vaccination sera determined as 2× above background of ovalbumin for representative Group 1 and 2 HA proteins

In vaccinated mouse sera (see Fig S3 for vaccination protocol), high titers of antibodies were detected against the homologous SI vaccine (Supplementary Fig. S4a, Supplementary Table S3), and post-vaccination sera competed with CR9114 for binding to the homologous SI (Fig. 2a–c). The Ph82-H3-SI post-vaccination sera showed the highest degree of competition compared to sera against the Hk68-H3-SI and Sh13-H7-SIs. In subsequent ELISAs, Hk68-H3-SI and Ph82-H3-SI post-vaccination sera had high titers of antibodies that bound recombinant A/Hong Kong/1/1968 (H3N2) HA, while the Sh13-H7-SI post-vaccination sera displayed lower titers **(**Fig. 3a **)**. All three SI induced comparable levels of antibodies against recombinant H7 HA **(**Fig. 3b **)**. Against HA expressed on virions, sera against the Hk68-H3-SI and Ph82-H3-SI contained high levels of binding antibodies to wild-type A/Hong Kong/1/1968 (H3N2), while both antisera showed limited or low level binding to the cold-adapted A/Anhui/1/2013 (H7N9) vaccine virus. In contrast, Sh13-H7-SI post-vaccination sera had high levels of antibodies that bound H7 virions, with only low level binding to H3 virions **(**Fig. 3c–d **)**. Antibody titers to whole virus were lower than to recombinant HA (rHA), possibly because regions of the HA stem have lower accessibility on virions. Notably, neither pre nor post-vaccination sera exhibited neutralizing activity against homologous or heterologous A/Hong Kong/1/1968 (H3N2), X-79 (H3N2), or A/Anhui/1/2013 (H7N9) viruses (Supplementary Table S4).

We next assessed the breadth of the antibody response to SI by comparing the ability of post-vaccination mouse sera to bind rHAs from a range of group 1 and 2 influenza viruses. As the SIs and rHAs contain His-tags, we verified that SI vaccination did not induce antibodies directed to this tag **(**Fig S4c **)**. Next, as shown in Fig. 3e, post-vaccination sera against all three SI exhibited binding to a broad range of HAs. Moreover, while the sera were raised against group 2 stem constructs, there were comparable end-point titers against both group 1 and 2 HA.

### Vaccination with group 2 SI protects mice from lethal homologous and intrasubtypic challenge

**Fig. 4:**
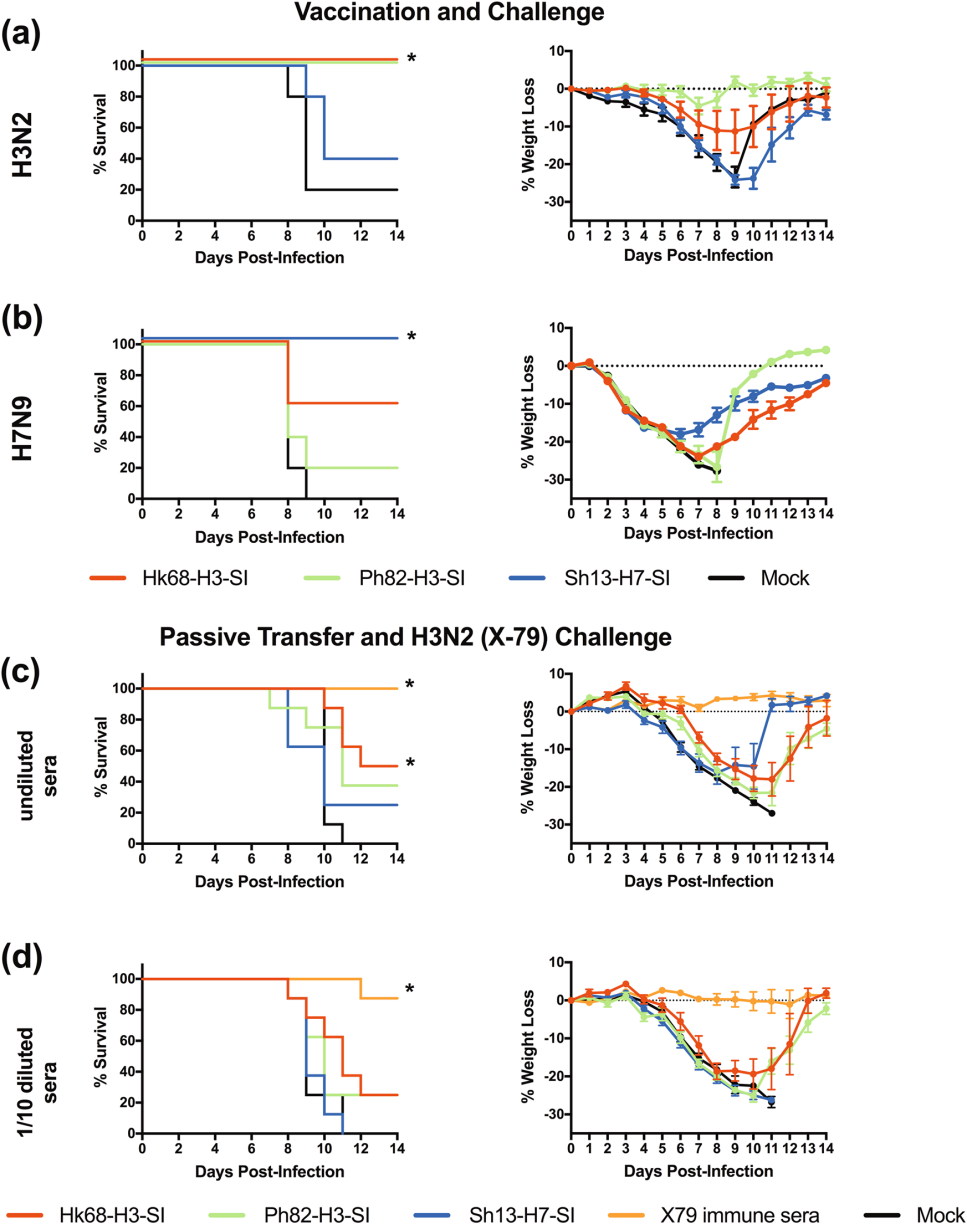
SI vaccination of mice protects against homologous challenge and provides partial protection against heterologous challenge, and passive transfer provides partial protection against homologous lethal challenge. To evaluate homologous and heterologous protection, SI-vaccinated mice were challenged with 10 × LD_50_ of X-79 (H3N2) or A/Anhui/1/2013 (H7N9). Panels **a** and **b** display survival (left) and weight loss (right) after X-79 (H3N2) and H7N9 influenza virus challenge, respectively. SI vaccination and challenge were performed once for each virus (*n* = 13/experimental group/virus challenge). To determine whether protection conferred by SI vaccination was antibody mediated, undiluted and 1/10 diluted post-vaccination sera were passively transferred to mice. An additional control group that received X-79 (H3N2) day 28 post-infection sera was also included. Twenty four hours after passive transfer, the mice were challenged with 10 x LD_50_ of X-79 (H3N2). Panels **c** and **d** display survival (left) and weight loss (right) for mice that received undiluted sera and 1/10 diluted sera, respectively. The passive transfer experiment was performed once with *n* = 8/group. * denotes significantly different from the mock-vaccinated groups (*p* < 0.05)

To assess the efficacy of SI vaccination, we challenged vaccinated mice with X-79 (H3N2) or A/Anhui/1/2013 (H7N9) virus. In the context of X-79 (H3N2) virus challenge, vaccination with the Ph82-H3-SI and Hk68-H3-SI’s completely protected against lethality (*p* < 0.005) and reduced weight loss **(**Fig. 4a **)**, while >60% of the Sh13-H7-SI and mock-vaccinated animals succumbed to infection. When vaccinated mice were challenged with A/Anhui/1/2013 (H7N9) virus, all mice lost >10% of their body weight; however, 100% of the Sh13-H7-SI-vaccinated animals survived (*p* = 0.01), while only 60, 20 and 0% of the Hk68-H3-SI, Ph82-H3-SI, and mock-vaccinated animals survived, respectively (Fig. 4b). When we evaluated viral replication in the lungs, on day 3 following H3N2 and H7N9 virus challenge, SI vaccination did not reduce viral load. On day 7 following homologous viral challenge, the Ph82-H3-SI and Sh13-H7-SI-vaccinated groups had a significantly (*p* = 0.028 and <0.001, respectively) lower viral load relative to mock-vaccinated animals indicating earlier clearance (Supplementary Fig. S5).

### Passive transfer confers partial protection in mice

To determine whether protection following SI-vaccination was antibody-mediated, we performed passive transfer experiments. Undiluted or 1:10 diluted post-vaccination sera was transferred to naïve mice, and the mice were challenged with 10 LD_50_ of X-79 (H3N2) virus. An additional group of mice received X-79 (H3N2) post-infection immune sera. As expected, the group of mice that received undiluted X-79 antisera were completely protected from lethality, and 90% of the mice that received a 1:10 dilution of this serum survived (Fig. 4c–d). Survival rates were 50 and 40% in mice that received passive transfer of undiluted Ph82-H3-SI and Hk68-H3-SI sera, respectively, and only 20% of both groups that received diluted sera survived. Only 20% of the mice that received undiluted Sh13-H7-SI antisera survived challenge, and there were no survivors when diluted sera was transferred (Fig. 4c–d). All of the mice, with the exception of those that received the X-79 (H3N2) post-infection antisera, lost >10% of their body weight (Fig. 4c–d). Thus, passive transfer of Hk68-H3-SI and Ph82-H3-SI post-vaccination antisera provided partial protection against lethal H3N2 virus challenge and this protection was dose dependent. However, the lack of complete protection in mice that received the homologous Ph82-H3-SI and intrasubtypic Hk68-H3-SI antisera suggests either a sub-optimal level of stem-directed antibody was transferred or SI-directed cellular immunity contribute to protection in vaccinated mice.

### Three doses of group 2 SI induce low levels of binding antibodies in ferrets

As group 2 stem targeting vaccination strategies have not been evaluated in the pre-clinical ferret model, we next evaluated the antibody response to group 2 SI vaccination in this species. We vaccinated ferrets according to a previously described regimen^21^ in which ferrets were given three adjuvanted vaccine doses 28 days apart (Supplementary Fig. S3). On day 26 following the 3rd dose of vaccine (day 82), the antibody response was evaluated. As a positive control, we included a group that received recombinant full-length H7 HA from A/Anhui/1/2013 (H7N9) with adjuvant. In comparison to mice, SI vaccination in ferrets induced lower titers of antibodies against the homologous SI antigens (Supplementary Fig. S4 and Supplementary Table S3), and in competitive binding assays with CR9114, post-vaccination ferret sera competed weakly with CR9114 for binding to the SI (Fig. 2d–f). Post-vaccination ferret sera also did not display neutralizing activity against A/Hong Kong1/1968 (H3N2), X-79 (H3N2), or A/Anhui/1/2013 (H7N9) (Supplementary Table S4); however, ferrets vaccinated with recombinant H7 HA (rH7) had moderate levels of H7N9-specific neutralizing antibody titers (1:60 mean neutralizing titer). We also evaluated the levels of binding antibodies directed against rHA protein and HA expressed on virions. Against recombinant A/Hong Kong/1/1968 (H3N2) HA, comparable titers were present in the rH7, Sh13-H7-SI and Ph82-H3-SI post-vaccination sera, while Hk68-H3-SI sera showed lower titers. Regardless of vaccine antigen, all of the post-vaccination sera had low titers of binding antibodies to wild-type A/Hong Kong/1/1968 (H3N2) virus (Supplementary Fig. S6a–b).

**Fig. 5:**
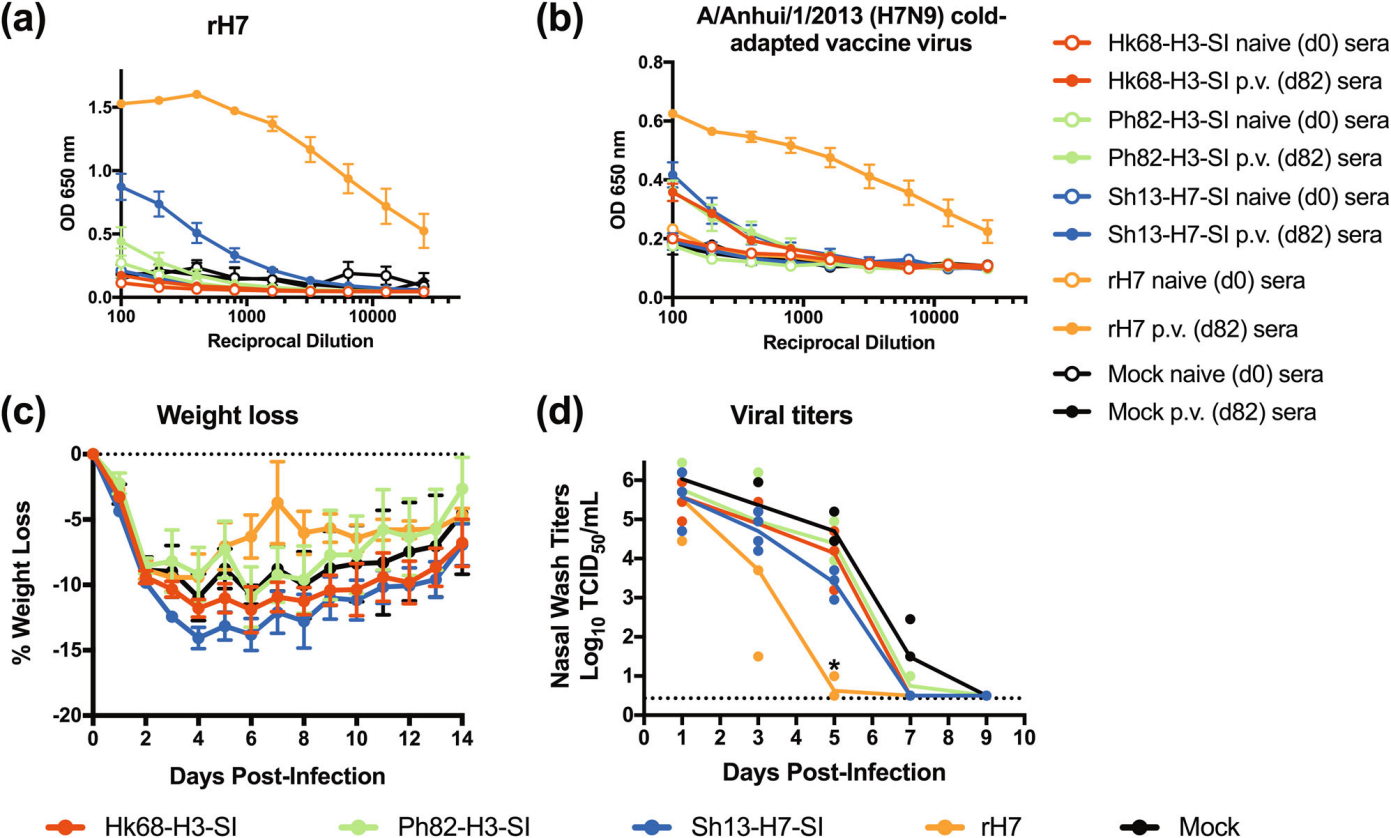
Sh13-H7-SI vaccination induces antibodies that bind H7 HA, but did not reduce clinical disease following robust H7N9 influenza challenge. Ferrets were given three doses of vaccine containing 50 µg of SI mixed with SAS adjuvant, 28 days apart. 26 days after receiving the third dose of vaccine, sera were collected and the ferrets were challenged 2 days later with 10^7^ TCID_50_ of A/Anhui/1/2013 (H7N9). A control group of ferrets was vaccinated with recombinant H7 HA from A/Anhui/1/2013 (H7N9). ELISA was performed using recombinant HA or live virus as coating antigens, **a** binding antibody titers against recombinant H7 HA from A/Anhui/1/2013 (H7N9) and **b** cold-adapted A/Anhui/1/2013 (H7N9) live-attenuated virus. Following virus challenge, ferrets were weighed daily and on days 1, 3, 5, 7 and 9, nasal washes were collected and titrated on MDCK cells. Panels **c** and **d** display percent weight loss and the nasal wash titers from all experimental groups, respectively. The ferret vaccination and challenge experiment was performed once with *n* = 4 group using equal numbers of male and female ferrets in each group. * significantly different (*p* < 0.0062) from mock-vaccinated animals. On day 5 the Sh13-H7-SI group had a non-significant 30-fold decrease in titers relative to the mock-vaccinated group (*p* = 0.161). Sera and body weights (*n* = 4/group) were expressed as mean ± s.e.m. In panel **d**, viral titers in nasal washes from individual animals are displayed and the line represents the mean titer at each time point

When ELISA was performed using H7 HA as the coating antigen, the rH7-vaccinated group had high titers of binding antibodies and an end-point titer was not achieved. Sh13-H7-SI-vaccinated ferrets had moderate levels of binding antibodies against H7 HA, while the Hk68-H3-SI and Ph82-H3-SI-vaccinated animals had low titers (Fig. 5a). Importantly, when titers against the H7 stalk were evaluated, the titers in the Sh13-H7-SI group were 16-fold higher than in the rH7 group, confirming that the SI preferentially induced stalk antibodies in ferrets (Supplementary Table S3). When sera were tested for binding to H7 HA expressed on H7N9 cold-adapted virus, endpoint titers in the rH7-vaccinated group were not reached. In contrast, all three SI-vaccinated groups had comparably low titers against H7 virions, indicating that despite detectable differences in titers against recombinant protein, the SI vaccines induced similar levels of antibodies capable of binding the H7 HA stem on the viral envelope **(**Fig. 5b **)**.

### SI vaccination did not prevent weight loss and caused a non-significant decrease in nasal wash titers following homologous H7N9 challenge in ferrets

To determine the efficacy of the group 2 SI vaccines, vaccinated ferrets were challenged with a robust dose of 10^7^ TCID_50_ of A/Anhui/1/2013 (H7N9) virus. After challenge, all ferrets lost weight, regardless of vaccination status with no significant differences between groups (Fig. 5c). None of the ferrets developed severe disease, and all ferrets had high titers of infectious virus in the nasal washes (Fig. 5d). On day 3 post-infection, relative to mock-vaccinated animals the rH7-vaccinated ferrets had decreased viral titers (*p* = 0.187), with a further reduction that became significant on day 5 (*p* = 0.0062). In the Sh13-H7-SI-vaccinated ferrets, there was a non-significant decrease of ~30 fold in viral titers on day 5 (*p* = 0.161)(Fig. 5d). For the Hk68-H3-SI and Ph82-H3-SI-vaccinated ferrets there were no reductions in viral titer in the nasal washes.

### Mapping of SI epitopes recognized by mouse and ferret post-vaccination sera

**Fig. 6:**
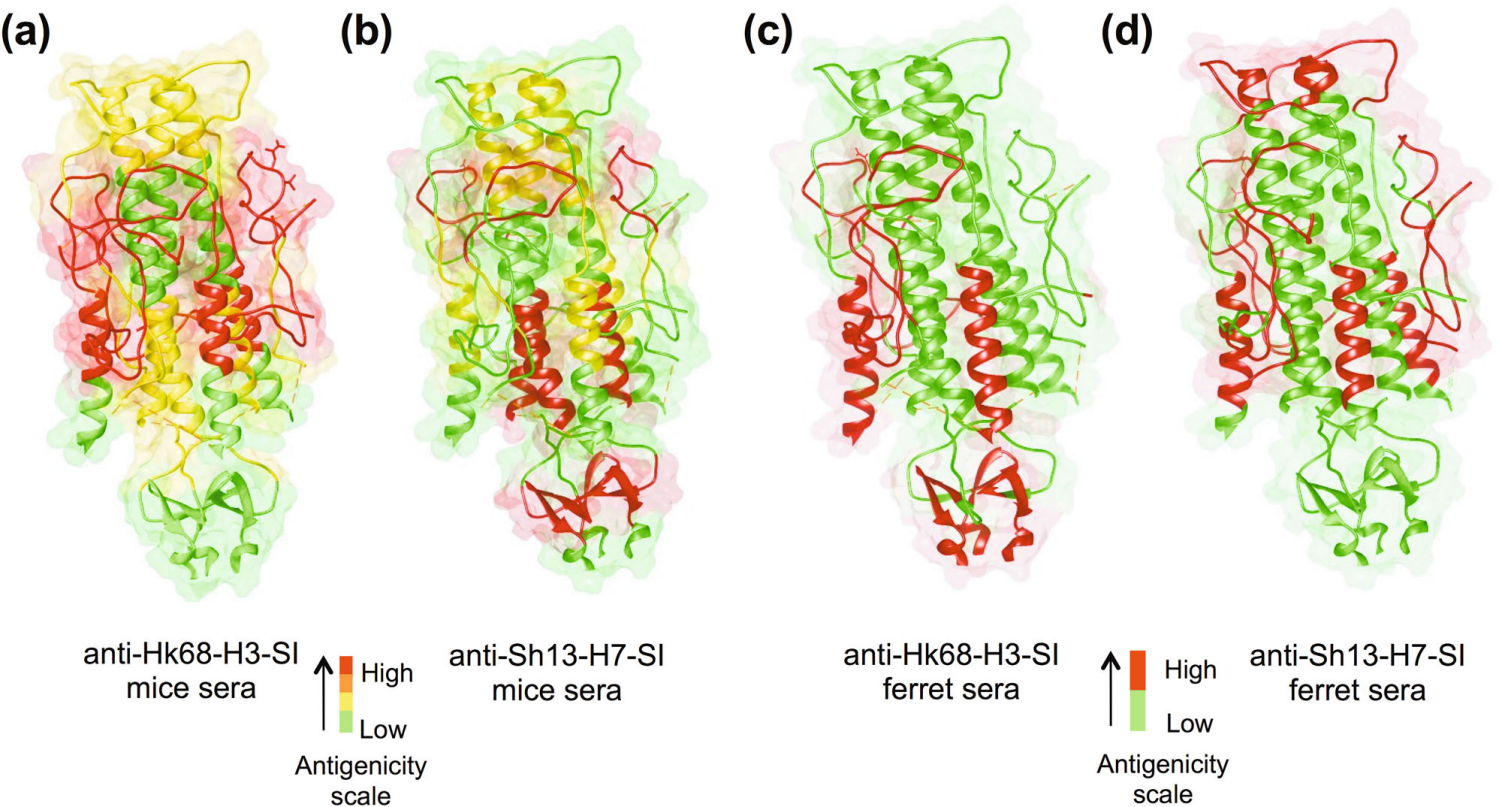
Epitope mapping of mouse and ferret antibody response in post-SI vaccination sera. To map the epitopes recognized by post-vaccination mouse and ferret sera, a library of N-terminal biotinylated peptides spanning the Hk68-H3-SI was designed and immobilized on streptavidin-coated plates. Mouse and ferret pre-vaccination and post-vaccination sera from the Hk68-H3-SI and Sh13-H7-SI-vaccinated groups were used to map the homologous and heterologous response. The antigenicity scale was defined based on the fold-change in signal intensity between pre and post-vaccination sera, and the response was mapped to a model of the Hk68-H3-SI. Panels **a** and **b** display antigenic maps of mouse sera against Hk68-H3-SI and Sh13-H7-SI, respectively, while panels **c** and **d** similarly display antigenic maps of ferret sera against Hk68-H3-SI and Sh13-H7-SI. Epitope mapping studies were performed in duplicate in three independent experiments. Results displayed are the mean values from the three independent replicates

To evaluate the antibody specificities induced by SIs, we performed epitope mapping with post-vaccination mouse and ferret sera. For the Hk68-H3-SI and Sh13-H7-SI mouse antisera, the response to peptides spanning solvent accessible patches of the CD-helix were moderate to high, while the response to the rest of the CD-helix was low, suggesting that both the Hk68-H3-SI and Sh13-H7-SI mimic the native, pre-fusion conformation of HA wherein the CD-helix is largely buried (Fig. 6a, b). As expected from the competitive binding experiments, the mouse sera against Hk68-H3-SI and Sh13-H7-SI span the epitope for CR9114, and the response to this epitope was high and moderate in the Hk68-H3-SI and Sh13-H7-SI sera, respectively. Epitope mapping of the post-vaccination ferret sera for Hk68-H3-SI and Sh13-H7-SI similarly demonstrated the presence of antibodies to peptides spanning the CR9114 epitope (Fig. 6c, d). The accessible HA1 region was immunogenic, but apart from the α-helix, the peptides covering the rest of HA2 region failed to elicit a detectable signal.

Importantly, post-vaccination mouse sera gave signal intensities as high as 15× above background for highly antigenic regions, while peak intensities for ferret sera were only 2× above background. Due to the limited degree of variation, the antigenic scale for ferret sera was scored as either high or low. Comparison of epitope mapping of mouse and ferret sera (Fig. 6a–d) indicated, the HA1 peptides and α-helix were immunogenic in both species; however, mice mounted an antibody response that spanned more of these regions and with greater overall intensity than ferrets.

## Discussion

Herein, we designed group 2 SIs and demonstrated that upon vaccination these antigens induced moderate titers of HA specific antibodies in mice but lower titers in ferrets. In mice, the SI conferred complete homologous and partial heterologous protection, and passive transfer studies suggest that vaccine efficacy is mediated in part by antibodies. However, in ferrets, vaccination did not prevent weight loss or significantly reduce viral titers, although Sh13-H7-SI-vaccinated animals had a non-significant ~30-fold reduction (*p* = 0.161) in nasal wash titers on day 5 following robust H7N9 challenge. Importantly, the mouse antisera to all three SI vaccines bound a broad range of group 1 and 2 HAs. This is a significant advance over other stem-targeted vaccine strategies as antibodies elicited by group 1 stem vaccines displayed minimal binding to the H3 and/or H7 (group 2) stalk region,^12,17,18,20,21^ and a chimeric HA vaccine bearing an H3 stem failed to induced antibodies capable of binding H1 HA.^15^

In comparison to our earlier studies, where H1 and H5 SI induced low titers of neutralizing antibodies against some H1 and H5 strains in mice,^20^ the group 2 SI did not induce detectable neutralizing activity; however, the efficacy of the group 2 SI vaccines is comparable to that of our group 1 SI vaccines.^18,20^ H1 and H5 SI vaccination completely protected mice from lethal homologous and intrasubtypic challenge, and conferred partial protection from heterologous protection. Taken together, it is likely that mechanisms such as antibody-dependent cell-mediated cytotoxicity, inhibition of HA maturation, or prevention of fusion of the viral and endosomal membranes play a role in protection.^31–33^

Using a chimeric HA vaccination strategy, complete homologous and heterologous protection has been shown against group 2 viruses in mice.^15^ In these studies, mice were primed with a DNA vaccine or influenza B virus bearing a chimeric influenza A HA. Animals were then given two doses of chimeric H3 HA vaccine or a single dose of chimeric HA followed by a dose of recombinant H3 HA. Vaccine efficacy was then assessed using a lethal H3N2 or H7N1 challenge.^15^ In the context of SI vaccines, it is conceivable that an additional boost with the SI or rHA would enhance protection against a heterologous H7 virus challenge.

In our study, SI-vaccinated ferrets developed antibodies that bound rHA and HA expressed on viral particles. When given a robust challenge with A/Anhui/1/2013 (H7N9) virus, ferrets were not protected from weight loss, and there were only modest changes in viral replication. Two group 1 stem vaccine approaches have been evaluated in ferrets;^21,34,35^ vaccination of ferrets with chimeric group 1 HAs induced high titers of non-neutralizing binding antibodies directed towards the H1 HA stalk. When these animals were challenged with 10^4^ pfu of H1N1pdm09 virus, ferrets had reduced virus titers in nasal washes on day 3.^34^ Using a different approach, when ferrets were vaccinated with H1 stem constructs on nanoparticles and challenged with 10^4^ TCID_50_ of a lethal highly pathogenic H5N1 virus, 4 of 6 animals survived the infection.^21^ An important difference between ferret studies with group 1 HA stem vaccines, and our study is that we used a 1000-fold higher challenge dose of virus. Challenge doses of 10^6^ or 10^7^ TCID_50_ of influenza viruses are commonly used to evaluate vaccine efficacy in ferrets.^36–39^ We specifically chose a dose of 10^7^ TCID_50_ because lower doses of A/Anhui/1/2013 (H7N9) cause minimal weight loss. It is possible that differences in viral replication would be more pronounced at a lower challenge dose and that heterologous vaccination may have conferred protection. Importantly, these studies highlight discrepancies between the approaches (i.e. challenge doses of 10^6^–10^7^ infectious units) for evaluating influenza vaccines that confer robust immunity compared to strategies (e.g. stem vaccines) that are expected to reduce disease severity but will not prevent infection.

In comparing the SI vaccines in mice and ferrets, we used comparable squalene-based oil-in-water adjuvants. Mice received two doses, while ferrets received three doses of vaccine. Following homologous virus challenge, mice were protected from severe disease and had reduced viral load, while ferrets were not protected from weight loss and had minimal changes in viral replication. Importantly, high titers of antibodies against purified virus were indicative of protection in mice, while antibody titers against purified virus were relatively low in ferrets. Thus, this measure may be an indicator of vaccine efficacy for stem-directed vaccines.

In epitope mapping studies, the signal intensity with ferret antisera was 5–8-fold lower and was focused on fewer epitopes than sera from mice. These findings explain the reduced efficacy of the group 2 SI vaccines in ferrets and highlight the need to evaluate stem vaccines in larger outbred species. Our findings are consistent with studies comparing the antibody response to serial infection in mice and ferrets,^40^ where ferrets had a narrower antibody response than mice following repeated infection with heterologous influenza viruses.

We are limited in our ability to compare our observations with previous studies evaluating group 1 HA stem vaccines in mice and ferrets because few investigators utilized similar approaches in both species. An exception is the nanoparticle-H1 stem vaccine, where mice and ferrets were vaccinated according to similar schedules.^21^ Upon lethal heterologous challenge with highly pathogenic H5N1 virus, mice were protected from lethality with no evidence of weight loss, while ferrets displayed some mortality (4 of 6 animals survived challenge) and all animals lost weight. This strategy resulted in good heterologous protection and it is possible that formulation of group 2 SI on nanoparticles or delivery in a live virus vector could enhance group 2 SI-mediated protection in ferrets. Importantly, our results indicate that successful development of a universal influenza vaccine, especially for group 2 HAs, will require improved magnitude and breadth of the antibody response.

In summary, we demonstrate vaccination with group 2 SI induced a non-neutralizing antibody response in mice and ferrets that contributed to complete homologous and partial heterologous protection in mice, and that may have enhanced viral clearance in ferrets. Strategies to broaden and enhance the immune response in ferrets are needed to advance SI vaccines, especially in the context of group 2 HAs. With improved immunogenicity, these vaccines have the potential to be used as universal influenza vaccines for the control of seasonal and pandemic influenza.

## Methods

### Design of group 2 SIs

To design the Hk68-H3-SI, using in-house PREDBURASA^41^ software, the contacts for each HA residue comprising the CR9114 epitope were identified and the hydrogen bond network and electrostatic interactions of the epitope residues were mapped. The HA stem interaction network centered along the epitope was extended outwards by iterative calculations until 90% of footprint for CR9114 and the “initiation” residues of the α-helix **(**Fig. 1c **)** were incorporated. The residues anterior to A44_2_, the N-terminus for the HA2 fragment, are included in Hk68-H3-SI and are critical to initiate the hydrogen bond network. Concomitantly, we extended the putative C-terminus of the HA2 fragment in Hk68-H3-SI to include interacting residues from the long central α-helices. These individual fragments were covalently “stitched” together and surface-exposed hydrophobic patches were resurfaced by introducing polar, hydrophilic mutations chosen by Rosetta Design^16,18,19,25^ **(**Fig. 1d **)**. Additionally, a synthetic trimerization motif, the globular, β-rich “foldon”, was appended to the C-terminus of the SI to promote oligomerization.^42^

The flipping-in of the foldon motif in HA stem constructs could be a consequence of a non-optimal linker length.^17,21^ Therefore, we calculated the distance between the C-terminal residue (M115_2_Q) of the HA stem fragment (37_2_–115_2_) and the N-terminal residue (*G*_1_) of foldon to determine the linker length wherein the following conditions are satisfied; coplanarity of the terminal (M115_2_Q, *G*_1_) residues, and superimposition of the 3-fold symmetry axes of HA and foldon (Fig. 1e **)**.

### Expression, purification, and characterization of SIs

Codon-optimized genes for SIs were synthesized (GenScript) and cloned into the bacterial expression vector pET-28a (+) (Novagen). The SIs were over-expressed in *E.coli* BL21(DE3) cells and purified.^18,19^ CD spectra, fluorescence spectroscopy, and size exclusion chromatography were measured or performed as previously described.^18,19^ The thermal stability of the SIs was determined by Nano-DSF (differential scanning fluorimetry) using a Prometheus NT.48 instrument (NanoTemper). SIs were evaluated at a concentration of 5 μM and thermal unfolding was performed at a rate of 1 °C/min in a range from 20–95 °C. Protein melting points (*T*_m_) were calculated according to the manufacturer’s protocol.

### Binding affinity measurements and competition binding using BLI

Binding affinity of the stem fragment immunogens (SIs) to CR9114, and competition between the post-vaccination sera and CR9114 IgG was determined by BLI using an Octet RED96 instrument (Pall ForteBio) as previously described.^20^

### Viruses and cell lines

Mouse-adapted X-79 (H3N2) (A/Philippines/2/1982 (H3N2): A/Puerto Rico/8/1934 (H1N1)) virus was amplified in the lungs of 4 week-old BALB/c mice (Taconic).^43^ A/Anhui/1/2013 (H7N9) was provided by the Centers for Disease Control (Atlanta, GA) and was grown in embryonated hen’s eggs. Virus stocks, nasal washes, and tissue homogenates were titrated on MDCK cells (ATCC), and virus titers were expressed as median tissue culture infectious dose (TCID_50_). MDCK cells were confirmed to be mycoplasma-free using the Mycoplasma Plus PCR Primer Set (Agilent Technologies).

### Mouse vaccination and challenge experiments

BALB/c mice (6-weeks old, female, *n* = 13/group) (Taconic Farms) were vaccinated with 20 μg of SI protein mixed 1:1 with Addavax adjuvant (InvivoGen) delivered via two 50 μL injections to each hind leg. Mice were vaccinated and boosted on day 28, and blood was collected on day 54. On day 56, mice were challenged with 10 LD_50_ of X-79 (H3N2) or A/Anhui/1/2013 (H7N9) virus, corresponding to 160 and 10^5^ TCID_50_/mouse, respectively, via intranasal inoculation. On days 3 and 7 pi, lung samples were collected (four mice/group) for viral titration, and the remaining animals were weighed daily for 14 days. Animals exhibiting 25% weight loss were humanely euthanized. Group size was determined based on the minimum number of animals needed to evaluate survival (*n* = 5/group) and viral replication in the lungs (*n* = 4/group/time point).

### Passive transfer experiments

For passive transfer studies, BALB/c mice (6-weeks old, female) (Taconic Farms) (*n* = 30/group) were vaccinated and boosted as above and blood was collected on day 56. To generate X-79 post-infection immune sera, an additional group of mice were infected with 0.1 LD_50_ of X-79 (H3N2) virus. Undiluted or 1/10 diluted sera (1 mL/mouse) was administered to naïve BALB/c mice (*n* = 8/dilution of antisera) via intraperitoneal injection. Twenty four hours later, mice were challenged with 10 LD_50_ of X-79 (H3N2) virus and weight loss and survival were monitored for 14 days. Group size for passive transfer studies was determined based upon the total amount of sera obtained after terminal bleed that would permit transfer of 1 mL of undiluted sera per mouse.

### Ferret vaccination and H7N9 virus challenge experiments

Twenty four-week-old ferrets (equal numbers of male and female animals, seronegative for circulating H1N1 and H3N2 viruses by HI assay) (Triple F Farms) (*n* = 4/group) were vaccinated with 50 μg of SI mixed 1:1 with Sigma Adjuvant System (SAS) adjuvant via intramuscular injection in the left hind leg. We utilized the SAS adjuvant in ferrets because it is formulated for larger animals and a Group 1 stem vaccine antigen^21^ formulated with it was previously shown to induce a protective antibody response. Three groups received Hk68-H3-SI, Ph82-H3-SI, and Sh13-H7-SI. Control groups each received 50 μg of full-length rHA from A/Anhui/1/2013 (H7N9) (Sino Biologicals), or adjuvant mixed with phosphate-buffered saline (PBS). Ferrets were vaccinated and boosted on days 0, 28 and 56, and were challenged intranasally on day 84 with 10^7^ TCID_50_ of A/Anhui/1/2013 (H7N9) virus. Ferrets were weighed daily to monitor weight loss and nasal washes were collected on days 1, 3, 5, 7, and 9 post-challenge. Group size (*n* = 4) was determined based on previous vaccine studies in ferrets^36,44^ and the housing limitations within the BSL3 facility. Upon evaluation of ferret sera by microneutralization assay (Supplementary Table S4), one ferret in the mock-vaccinated group was found to have low levels of pre-existing antibody titers against H3N2 viruses. This animal was removed from all analyses.

### Biocontainment and animal protocols

Vaccination and X-79 (H3N2) virus challenge were conducted under BSL2 conditions. All work with A/Anhui/1/2013 (H7N9) was conducted in enhanced BSL3 laboratories at the National Institutes of Health (NIH). For both the mouse and ferret studies, animals were not randomized and the studies were not blinded. All animal studies were approved by the NIH Animal Care and Use Committee, and were performed in accordance with relevant regulations and guidelines.

### Quantification of post-vaccination antibody titers against SI, rHA, and whole virus

Post-vaccination binding antibody titers to SI were determined by ELISA as previously described.^18^ Binding antibody titers against full-length rHA and purified viruses were also determined by ELISA. Briefly, 96-well plates (Nalgene) were coated with 50 ng of rHA (A/Hong Kong/1/1968 (H3N2), or A/Anhui/1/2013 (H7N9) (Sino Biologicals)), blocked with 10% bovine serum albumin in PBS-Tween 20 (0.05%)(PBS-T) or 5% skim milk powder in PBS-T for mouse and ferret sera, respectively. Dilutions of heat-inactivated sera were incubated on the plates for 2 h at room temperature, followed by washing with PBS-T and incubation for 1 h with HRP labeled goat-anti mouse IgG polyclonal antibody (Abcam) or goat-anti ferret IgG polyclonal antibody (Alpha Diagnostics). After rinsing, plates were developed with SureBlue TMB Microwell Peroxidase Substrate and TMB BlueSTOP Solution (KPL), or p-nitrophenyl phosphate substrate, and the OD was measured at 650 nm or 405 nm, respectively, on a SpectraMax plate reader (Molecular Devices).

To measure binding to whole virus, the protocol was modified such that 128 HA units of virus in PBS was added to each well. The plates were incubated overnight at 4 °C, blocked, rinsed, and developed as above, with the exception that incubation of the mouse and ferret sera was performed at 37 °C.

### Determining the antigenic breadth of the SIs

ELISA was used to determine the cross-reactive antibody titers elicited post-vaccination by the SIs. Assays were performed as previously described^18^ using a panel of Group 1 and Group 2 rHA proteins **(**Fig. 3e **)** (Sino Biological).

### Peptide epitope mapping

A set of N-terminal biotinylated peptides (15mer) spanning Hk68-H3-SI with an overlap of ten residues were synthesized by Pepscan. For epitope mapping by ELISA, 96-well Maxisorp plates were coated with streptavidin at 5 μg/ml and incubated overnight at 4 °C. The plates were washed with PBST, and a 100-fold molar excess of peptide (dissolved in 0.2 M sodium carbonate/bicarbonate buffer, pH 9.4) was added to each well and incubated overnight at 4 °C. The plates were washed and then blocked with PBST + 1%BSA. Next, 50 μL of pre-vaccination or post-vaccination mouse (1:100 dilution) or ferret (1:20 dilution) sera was added to each well and incubated for 2 h at room temperature. The plates were washed with PBST and 50 μL of ALP-conjugated goat anti-mouse antibody (Sigma) (1:10000 dilution) or HRP-conjugated goat anti-ferret secondary antibody (Alpha Diagnostics) (1:10000 dilution) was added and incubated for 1 h at room temperature. The plates were subsequently developed as described for ELISA against SI. The antigenicity of each peptide was determined as the ratio between the signals obtained from the post-vaccination and pre-vaccination sera.

### Statistical analysis

Groups of mice and ferrets were compared at each time point using a one-way non-parametric Kruskal–Wallis test with post hoc Dunn’s multiple comparison test against the mock-vaccinated group. Prior to performing non-parametric analyses, variance between groups was evaluated using a Brown–Forsythe test and were not significantly different between groups. Survival curves were compared using a Mantel–Cox test with post-hoc pairwise comparisons using a Bonferroni correction. All analyses were performed using Prism 7 GraphPad software with *p* < 0.05 considered significant.

### Data availability

Data supporting the findings are available from the corresponding authors upon reasonable request.

## Electronic supplementary material

Supplemental Tables 1-4

Supplemental Figures 1-6

## References

[1] WHO. *2016 Influenza (Seasonal) fact sheet* http://www.who.int/mediacentre/factsheets/fs211/en/ (2016).

[2] Taubenberger JK, Morens DM (2006). 1918 Influenza: the mother of all pandemics. Emerg. Infect. Dis..

[3] Luke, C. J., Lakdawala, S. S. & Subbarao, E. K. in *Vaccines*, Vol. 6 (eds S. A. Plotkin, W. A. Orenstein, & P. A. Offit) Ch. 18, 294–311 (Elsevier, Philadelphia, 2013).

[4] Wu Y, Wu Y, Tefsen B, Shi Y, Gao GF (2014). Bat-derived influenza-like viruses H17N10 and H18N11. Trends Microbiol..

[5] Skehel JJ, Wiley DC (2000). Receptor binding and membrane fusion in virus entry: the influenza hemagglutinin. Annu. Rev. Biochem..

[6] Corti D, Lanzavecchia A (2013). Broadly neutralizing antiviral antibodies. Annu. Rev. Immunol..

[7] Corti D (2010). Heterosubtypic neutralizing antibodies are produced by individuals immunized with a seasonal influenza vaccine. J. Clin. Invest..

[8] Dreyfus C (2012). Highly conserved protective epitopes on influenza B viruses. Science.

[9] Miller MS (2013). Neutralizing antibodies against previously encountered influenza virus strains increase over time: a longitudinal analysis. Sci. Transl. Med..

[10] Throsby M (2008). Heterosubtypic neutralizing monoclonal antibodies cross-protective against H5N1 and H1N1 recovered from human IgM+memory B cells. PLoS. One..

[11] Van, T. D., Tran, N., Le, L. & Eisenhaber, F. A Perspective on rational designs of a hemagglutinin based universal influenza vaccine. *Curr. Pharm. Des.* 3547–3554 (2016). 24308491

[12] Krammer F, Pica N, Hai R, Margine I, Palese P (2013). Chimeric hemagglutinin influenza virus vaccine constructs elicit broadly protective stalk-specific antibodies. J. Virol..

[13] Steel, J. et al. Influenza virus vaccine based on the conserved hemagglutinin stalk domain. *MBio*. 10.1128/mBio.00018-10 (2010). 10.1128/mBio.00018-10 PMC2912658 20689752

[14] Wei CJ (2010). Induction of broadly neutralizing H1N1 influenza antibodies by vaccination. Science.

[15] Margine I (2013). Hemagglutinin stalk-based universal vaccine constructs protect against group 2 influenza A viruses. J. Virol..

[16] Bommakanti G (2012). Design of Escherichia coli-expressed stalk domain immunogens of H1N1 hemagglutinin that protect mice from lethal challenge. J. Virol..

[17] Impagliazzo A (2015). A stable trimeric influenza hemagglutinin stem as a broadly protective immunogen. Science.

[18] Mallajosyula VV (2014). Influenza hemagglutinin stem-fragment immunogen elicits broadly neutralizing antibodies and confers heterologous protection. Proc. Natl. Acad. Sci. USA.

[19] Mallajosyula VV (2015). Hemagglutinin sequence conservation guided stem immunogen design from influenza A H3 subtype. Front. Immunol..

[20] Valkenburg SA (2016). Stalking influenza by vaccination with pre-fusion headless HA mini-stem. Sci. Rep..

[21] Yassine HM (2015). Hemagglutinin-stem nanoparticles generate heterosubtypic influenza protection. Nat. Med..

[22] Krammer F, Pica N, Hai R, Tan GS, Palese P (2012). Hemagglutinin stalk-reactive antibodies are boosted following sequential infection with seasonal and pandemic H1N1 influenza virus in mice. J. Virol..

[23] Corti D (2011). A neutralizing antibody selected from plasma cells that binds to group 1 and group 2 influenza A hemagglutinins. Science.

[24] Julien JP, Lee PS, Wilson IA (2012). Structural insights into key sites of vulnerability on HIV-1 Env and influenza HA. Immunol. Rev..

[25] Bommakanti G (2010). Design of an HA2-based Escherichia coli expressed influenza immunogen that protects mice from pathogenic challenge. Proc. Natl. Acad. Sci. USA.

[26] Krammer F, Palese P (2015). Advances in the development of influenza virus vaccines. Nat. Rev. Drug Discov..

[27] Lu Y, Welsh JP, Swartz JR (2014). Production and stabilization of the trimeric influenza hemagglutinin stem domain for potentially broadly protective influenza vaccines. Proc. Natl. Acad. Sci. USA.

[28] Ekiert DC (2011). A highly conserved neutralizing epitope on group 2 influenza A viruses. Science.

[29] Friesen RH (2014). A common solution to group 2 influenza virus neutralization. Proc. Natl. Acad. Sci. USA.

[30] Jia, B. & Jeon, C. O. High-throughput recombinant protein expression in Escherichia coli: current status and future perspectives. *Open Biol*10.1098/rsob.160196 (2016). 10.1098/rsob.160196 PMC5008019 27581654

[31] Brandenburg B (2013). Mechanisms of hemagglutinin targeted influenza virus neutralization. PLoS One.

[32] DiLillo DJ, Palese P, Wilson PC, Ravetch JV (2016). Broadly neutralizing anti-influenza antibodies require Fc receptor engagement for in vivo protection. J. Clin. Invest..

[33] DiLillo DJ, Tan GS, Palese P, Ravetch JV (2014). Broadly neutralizing hemagglutinin stalk-specific antibodies require FcgammaR interactions for protection against influenza virus in vivo. Nat. Med..

[34] Krammer F (2014). Assessment of influenza virus hemagglutinin stalk-based immunity in ferrets. J. Virol..

[35] Nachbagauer R (2015). Hemagglutinin stalk immunity reduces influenza virus replication and transmission in ferrets. J. Virol..

[36] Chen GL (2014). Evaluation of three live attenuated H2 pandemic influenza vaccine candidates in mice and ferrets. J. Virol..

[37] Houser KV, Pearce MB, Katz JM, Tumpey TM (2013). Impact of prior seasonal H3N2 influenza vaccination or infection on protection and transmission of emerging variants of influenza A(H3N2)v virus in ferrets. J. Virol..

[38] Pushko P (2015). Recombinant H7 hemagglutinin forms subviral particles that protect mice and ferrets from challenge with H7N9 influenza virus. Vaccine.

[39] Wong SS (2014). A single dose of whole inactivated H7N9 influenza vaccine confers protection from severe disease but not infection in ferrets. Vaccine.

[40] Nachbagauer R (2017). Defining the antibody cross-reactome directed against the influenza virus surface glycoproteins. Nat. Immunol..

[41] Sharma D (2005). Protein minimization of the gp120 binding region of human CD4. Biochemistry.

[42] Guthe S (2004). Very fast folding and association of a trimerization domain from bacteriophage T4 fibritin. J. Mol. Biol..

[43] Chen KS, Quinnan GV (1988). Induction, persistence and strain specificity of haemagglutinin-specific secretory antibodies in lungs of mice after intragastric administration of inactivated influenza virus vaccines. J. Gen. Virol..

[44] Baz M (2015). A single dose of an avian H3N8 influenza virus vaccine is highly immunogenic and efficacious against a recently emerged seal influenza virus in mice and ferrets. J. Virol..

