# Supplementary material for: Protective efficacy of influenza group 2 hemagglutinin stem-fragment immunogen vaccines
Source: NPJ Vaccines. 2017 Dec 15;2:35. doi: 10.1038/s41541-017-0036-2 (PMC5732283; doi:10.1038/s41541-017-0036-2)
Supplement: Supplementary file 1 — Supplemental Tables 1-4 [file 41541_2017_36_MOESM1_ESM.docx]

**Table S1. Percent amino acid identify of the viral HA and stem immunogens with HA from the challenge virus**

| **Influenza A group 2 HA** | | **% amino acid identity with the challenge virus** | |
| --- | --- | --- | --- |
|  |  | **X-79 (H3N2)**  **[A/Philippines/2/1982]** | **A/Anhui/1/2013 (H7N9)** |
| **Viral HA** | A/Hong Kong/1/1968 (H3N2)^a^ | 91.5 | 47.9 |
|  | A/Philippines/2/1982 (H3N2)^a^ | 100 | 47.4 |
|  | A/Shanghai/2/2013 (H7N9)^a^ | 47.4 | 98.2 |
| **Stem Immunogen** | Hk68-H3-SI^b^ | 92.6 | 56.3 |
|  | Ph82-H3-SI^b^ | 94.1 | 56.3 |
|  | Sh13-H7-SI^b^ | 57.0 | 92.6 |

^a^ Full-length HA sequences were obtained from the NCBI influenza database.

^b^ Alignment was performed with HA-SI sequences containing all the engineered mutations, without the linker and ‘foldon’ motif.

**Table S2. Binding affinity of CR9114 for group 2 stem-fragment immunogens determined by biolayer interferometry (BLI).**

| **Analyte** | ***k*_on_ (1/Ms)** | ***k*_off_ (1/s)** | ***K*_D_ (M)** |
| --- | --- | --- | --- |
| **Hk68-H3-SI** | 5.24±3.84x10^4^ | 4.73±0.59x10^-5^ | 1.54±1.43x10^-9^ |
| **Ph82-H3-SI** | 1.22±0.37x10^4^ | 4.28±2.79x10^-5^ | 3.30±1.27x10^-9^ |
| **Sh13-H7-SI** | 1.93±1.48x10^4^ | 1.31±0.06x10^-3^ | 1.27±1.42x10^-7^ |

Results are expressed as mean ± S.D. of three independent experiments.

**Table S3. Homologous stem-fragment immunogen directed antibody titers in pre and post-vaccination mouse and ferret sera.**

|  | **Mouse SI Antibody Titers** | | **Ferret SI Antibody Titers** | |
| --- | --- | --- | --- | --- |
|  | Days Post-Vaccination | | Days Post-Vaccination | |
| **Group** | Day 0 | Day 54* | Day 0 | Day 82** |
| **HK68-H3-SI** | <1:100 | 1:409600 | 1:100 | 1:25600 |
| **Ph82-H3-SI** | <1:100 | 1:409600 | 1:100 | 1:25600 |
| **Sh13-H7-SI** | <1:100 | 1:409600 | 1:400 | 1:25600 |
| **rH7***** | n/a | n/a | 1:400 | 1:1600 |

Lower limit of detection was 1:100 for mouse and ferret ELISA. The titer was determined as the dilution at which the signal from sera to immobilized antigen was ≥ 2X signal for ovalbumin, and above an absolute value of 0.1.

* 26 days after 2 doses of vaccine

** 28 days after 3rd dose of vaccine

*** antibody titers for this group were evaluated against the Sh13-H7-SI. n/a is denoted because there was no rH7 vaccinated group in the mouse experiments

**Table S4. Neutralizing titers in SI vaccinated mice and ferrets**

| **Species** | **Vaccine Antigen** | **Post-vaccination^a^ neutralizing antibody titers against indicated viruses^b^** | | |
| --- | --- | --- | --- | --- |
|  |  | **A/Hong Kong/1/1968 (H3N2)** | **X-79 (H3N2)**  **[A/Philippines/2/1982]** | **A/Anhui/1/2013 (H7N9)** |
| **Mouse** | Hk68-H3-SI | <10 | <10 | <10 |
|  | Ph82-H3-SI | <10 | <10 | <10 |
|  | Sh13-H7-SI | <10 | <10 | <10 |
|  | Mock | <10 | <10 | <10 |
| **Ferret** | Hk68-H3-SI | <10 | <10 | <10 |
|  | Ph82-H3-SI | <10 | <10 | <10 |
|  | Sh13-H7-SI | <10 | <10 | <10 |
|  | rH7 | <10 | <10 | **60** |
|  | Mock^c^ | <10 | <10 | <10 |

**^a^**Mouse sera were collected on day 54 (26 days following the 2^nd^ dose of vaccine), and ferret sera were collected on day 82 (26 days after receiving a third dose of vaccine).

**^b^**Neutralizing titers are expressed as geometric mean titers and were performed as described previously^1^

^c^One ferret in the mock vaccinated group was found to have low titers of detectable neutralizing titers against H3N2 viruses in the pre-vaccination (d0) sera. This animal was removed from all analyses.

**REFERENCES FOR SUPPLEMENTAL MATERIALS**

1 Chen, G. L. *et al.* Evaluation of replication and cross-reactive antibody responses of H2 subtype influenza viruses in mice and ferrets. *J Virol* **84**, 7695-7702, doi:10.1128/JVI.00511-10 (2010).
