# Supplementary material for: Protective efficacy of influenza group 2 hemagglutinin stem-fragment immunogen vaccines
Source: NPJ Vaccines. 2017 Dec 15;2:35. doi: 10.1038/s41541-017-0036-2 (PMC5732283; doi:10.1038/s41541-017-0036-2)
Supplement: Supplementary file 2 — Supplemental Figures 1-6 [file 41541_2017_36_MOESM2_ESM.pdf]

Hk68-H3-SI (derived from A/Hong Kong/1/1968 (H3N2))

TLVKTITDDQIEVTNATELVQSS**GSAG**NDKPFQNTNKRTSGASPKYVKQNTLKLATGNRN**GSAGSA**
DLKSTQAAIDQINGKLNRVIEKTNEK***D***HQIEKEFSE***D***EGRIQDLEKYVEDTKIDLWSYNAELLVAL
ENQHTIDLTDSEQ**GG***GYIPEAPRDGQAYVRKDGEWVLLSTFL*

Ph82-H3-SI (derived from A/Philippines/2/1982 (H3N2))

TLVKTITNDQIEVTNATELVQSS**GSAG**NDKPFQNTNKNTHGASPRYVKQNTLKLATGQRN**GSAGSA**
DLKSTQAAIDQINGKLNRVIEKTNEK***D***HQIEKEFSE***D***EGRIQDLEKYVEDTKIDLWSYNAELLVAL
ENQHTIDLTDSEN**GGS***GYIPEAPRDGQAYVRKDGEWVLLSTFL*

Sh13-H7-SI (derived from A/Shanghai/1/2013(H7N9))

TKVNTLTERGVEVTNATETVERT**GSA**SNLPFQNNDSRASGKSPRYVKQRSLLLATGNKN**GSAGSA**D
YKSTQSAIDQITGKLNRLIEKTNQQFE***D***IDNE***D***TETEKQIGNVINWTRDSITEVWSYNAELLVAME
NQHTIDLADSEQ**GG***GYIPEAPRDGQAYVRKDGEWVLLSTFL*

Figure S1: Sequence of the designed SIs. The immunogens were designed from full-length HA sequences obtained from the National Center for Biotechnology Information Influenza Virus Database [H3N2 A/Hong Kong/1/1968 (AAK51718.1), H3N2 A/Philippines/2/1982 (ADJ41805.1), and H7N9 A/Shanghai/1/2013 (AGL44438.1)]. Mutations (underlined) introduced to resurface the exposed hydrophobic patches were chosen by Rosetta Design. Residues in the loop were mutated to Asp (bold and italics) to destabilize the low-pH conformation of HA. Cys305$_1$ was mutated to Ser to prevent the formation of incorrect, intermolecular disulfide bonds. The HA fragments were connected by soluble, flexible linkers (bold). A synthetic trimerization motif, ‘foldon’ (italics), was appended to the C-terminus of the designed SIs.

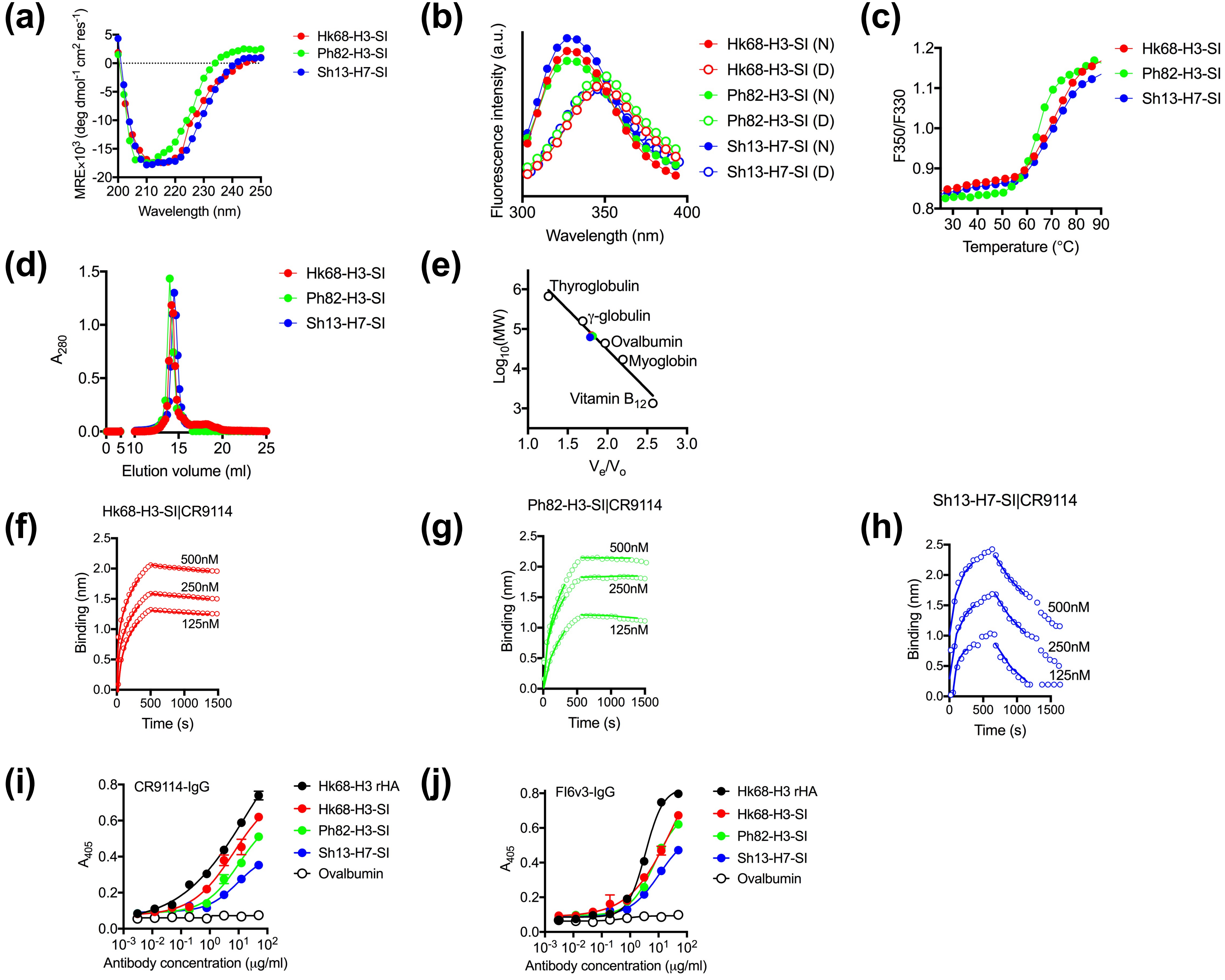
(a)
MRE×10^3 (deg dmol^-1 cm^2 res^-1)
Wavelength (nm)
Hk68-H3-SI
Ph82-H3-SI
Sh13-H7-SI
(b)
Fluorescence intensity (a.u.)
Wavelength (nm)
Hk68-H3-SI (N)
Hk68-H3-SI (D)
Ph82-H3-SI (N)
Ph82-H3-SI (D)
Sh13-H7-SI (N)
Sh13-H7-SI (D)
(c)
F350/F330
Temperature (°C)
(d)
A280
Elution volume (ml)
(e)
Log10(MW)
Thyroglobulin
γ-globulin
Ovalbumin
Myoglobin
Vitamin B12
Ve/Vo
(f)
Hk68-H3-SI|CR9114
(g)
Ph82-H3-SI|CR9114
(h)
Sh13-H7-SI|CR9114
Binding (nm)
Time (s)
500nM
250nM
125nM
(i)
CR9114-IgG
(j)
FI6v3-IgG
A405
Antibody concentration (μg/ml)
Hk68-H3 rHA
Hk68-H3-SI
Ph82-H3-SI
Sh13-H7-SI
Ovalbumin

Figure S2

Figure S2. Biophysical and biochemical characterization of the SIs. (a) The CD spectra of all the purified SIs in the far-UV region (200-250nm) was characteristic of a well-folded, −helical protein. **(b)** The intrinsic tryptophan fluorescence emission maxima of the SIs under native (N) and denatured (D) conditions were measured. The red-shift in emission maxima upon denaturation with GdmCl indicates a well-packed conformation with buried hydrophobic residues under native conditions. For panels A and B, the spectra were averaged over ten consecutive scans and corrected for buffer signals (PBS, pH 7.4) under similar conditions. **(c)** The thermal stability of SIs was estimated by differential scanning fluorimetry (DSF). The ratio of the intrinsic protein fluorescence measured in-parallel at 330nm and 350nm as a function of temperature is shown. The high transition-midpoints (T_m); Hk68-H3-SI (72ºC), Ph82-H3-SI (66ºC), and Sh13-H7-SI (71ºC) of thermal unfolding for SIs suggests that the designed proteins are resistant to thermal stress. The represented unfolding traces were averaged over three independent readings. **(d)** The oligomeric state of the SIs in solution was determined by analytical gel-filtration chromatography under non-denaturing conditions (PBS, pH 7.4) using a Superdex-200 column. The SIs eluted exclusively as trimers in solution. The lack of higher-order aggregates in solution further suggests that resurfacing exposed hydrophobic patches mitigates aggregation. **(e)** The column was calibrated using standards spanning a broad range of molecular weights (open circles) under similar conditions (PBS, pH 7.4). The data-points corresponding to the elution volumes of the SIs is represented by solid circles. For panels d and e, purified proteins were analyzed in three independent runs and the panels display traces from a representative run. Binding traces of CR9114 (IgG) to the SIs; **(f)** Hk68-H3-SI, **(g)** Ph82-H3-SI, and **(h)** Sh13-H7-SI as determined by biolayer interferometry using Octet. Panels **(i)** and **(j)** display direct ELISA binding profiles for the conformation specific bnAbs CR9114 and FI6v3, respectively, against the three SI and recombinant HA from A/Hong Kong/1/1968 (H3N2). Binding assays were performed in three independent experiments and shown are representative traces from one experiment.

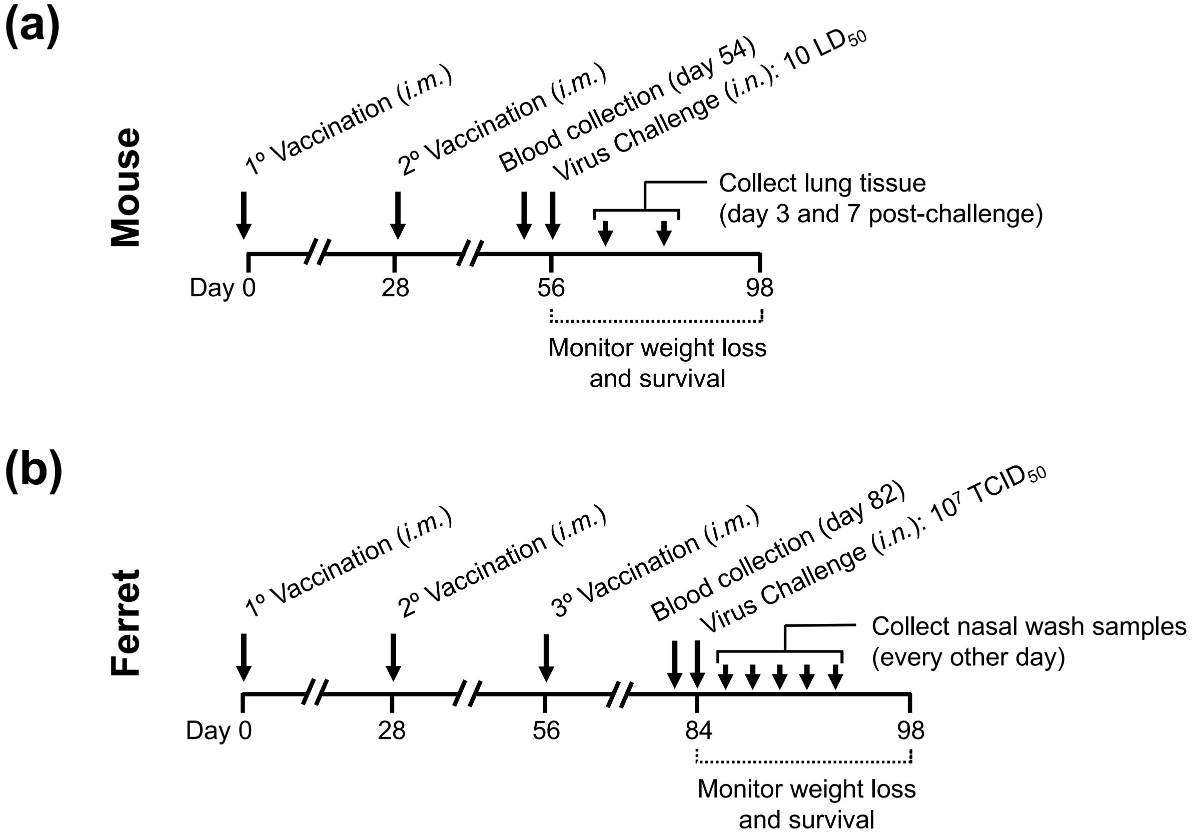

Figure S3. Vaccination and challenge protocols for mouse and ferret studies. Animals were given intramuscular vaccinations consisting of SI mixed with adjuvant. Panels **(a)** and **(b)** display vaccination regiment for mice and ferrets, respectively. Mice were vaccinated with 20 g of SI mixed with Addavax adjuvant and were given two doses of vaccine. Ferrets were vaccinated with 50 g of SI mixed with Sigma Adjuvant System (SAS) and were given three doses of vaccine. Mice and ferrets were challenged *intranasally* with 10 LD_{50} and 10^7 $TCID_{50}$, respectively, and were monitored for weight loss and survival. On day 3 and 7 post-challenge, mouse lung tissues (n=4) were collected for viral titration, and in the ferret studies on days 1, 3, 5, 7, and 9 post-challenge nasal washes samples were collected from all animals to determine viral titers in the nose.

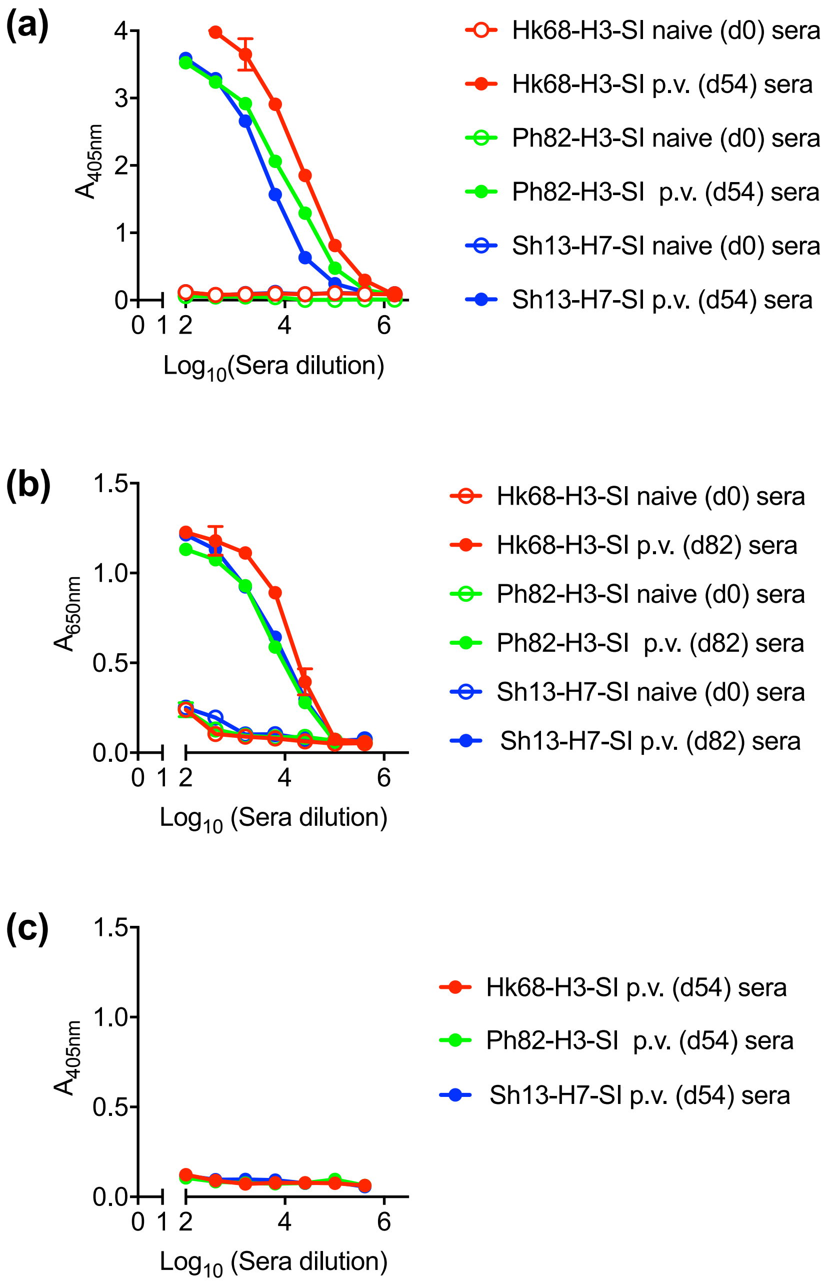

Figure S4. SI vaccination of mice and ferrets induces high titers of stem antibodies. Binding antibody titers against SI were determined by ELISA in naive (d0) and post-vaccination (p.v.) mouse and ferret sera. Binding titers in **(a)** mouse and **(b)** ferret pre and post-vaccination sera against homologous SI. In panel **(c)** binding antibody titers against the His-tag were measured by ELISA in post-vaccination sera using His-tagged HIV-1 env protein. ELISA were performed twice in triplicate on pooled mouse sera and results are expressed as mean ± S.D.

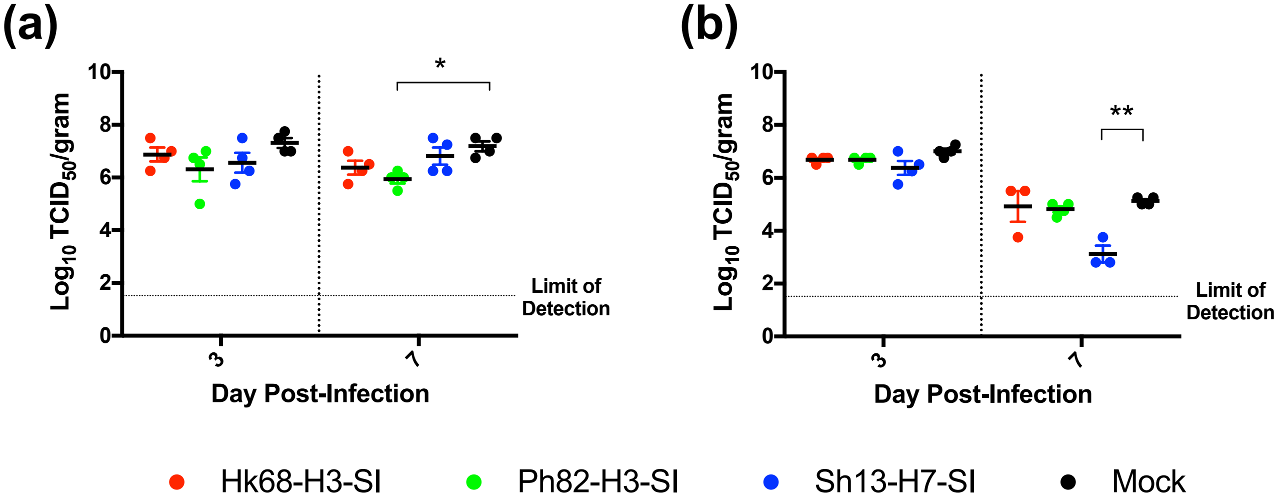

Figure S5. Homologous SI vaccination reduces viral load in the lungs of mice. On day 3 and 7 post-viral challenge of SI vaccinated mice (n=4/group), lung samples were collected, homogenized, and titrated on MDCK cells. The results are expressed as $TCID_{50}$/gram of tissue. SI vaccination and challenge were performed once for each virus (n=4/time point/experimental group/virus challenge). Panel **(a)** and **(b)** show lung titers after X-79 (H3N2) and A/Anhui/1/2013 (H7N9) challenge in separate vaccination and challenge experiments, respectively. * p = 0.0275 ** p = <0.001.

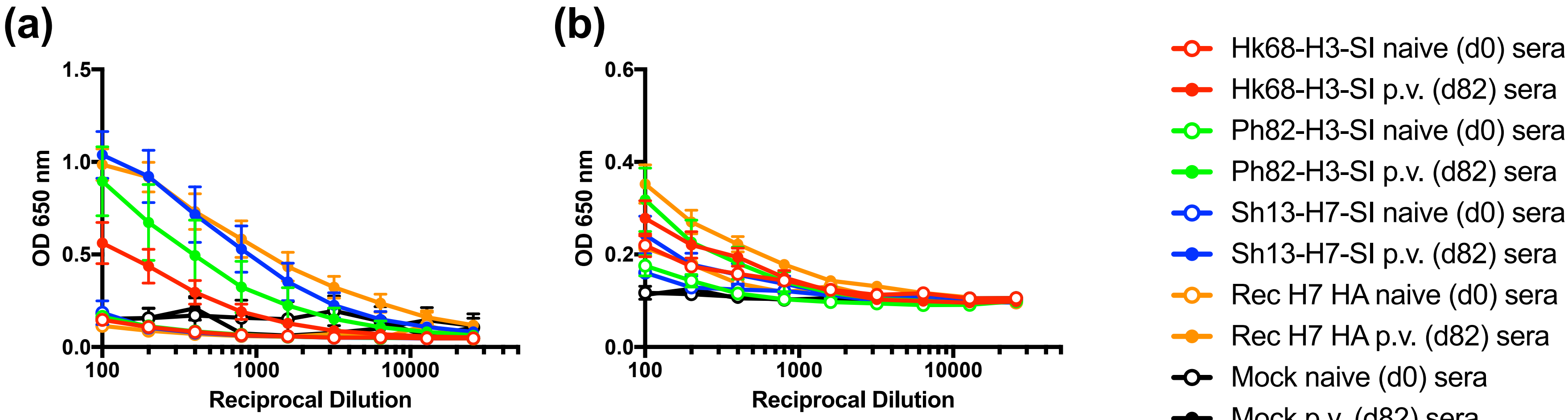

Figure S6. Ferrets vaccinated with SI develop non-neutralizing antibodies that bind recombinant H3 HA proteins and H3 HA on viral particles. Binding antibody titers against recombinant HA were determined for sera from all groups of vaccinated ferrets. Shown in **(a)** and **(b)** are ELISA titers against recombinant H3 HA from A/Hong Kong/1/1968 (H3N2) and wild-type A/Hong Kong/1/1968 (H3N2) virus, respectively. ELISA were optimized with pooled ferret sera (due to limited volume of sera) and subsequently, each animal was assayed independently (n=4/group). Results were expressed as mean ±s.e.m.
